# Characterization of the Apoptotic and Antimicrobial Activities of Two Initiator Caspases of Sea Cucumber *Apostichopus japonicus*

**DOI:** 10.3390/genes15050540

**Published:** 2024-04-25

**Authors:** Hanshuo Zhu, Zihao Yuan, Hang Xu, Li Sun

**Affiliations:** 1 CAS and Shandong Province Key Laboratory of Experimental Marine Biology, Institute of Oceanology, Center for Ocean Mega-Science, Chinese Academy of Sciences, Qingdao 266404, China; 2 Laboratory for Marine Biology and Biotechnology, Qingdao Marine Science and Technology Center, Qingdao 266237, China; 3 College of Marine Sciences, University of Chinese Academy of Sciences, Qingdao 266404, China

**Keywords:** *Apostichopus japonicus*, caspase, apoptosis, antimicrobial

## Abstract

Caspase (CASP) is a protease family that plays a vital role in apoptosis, development, and immune response. Herein, we reported the identification and characterization of two CASPs, AjCASPX1 and AjCASPX2, from the sea cucumber *Apostichopus japonicus*, an important aquaculture species. AjCASPX1/2 share similar domain organizations with the vertebrate initiator caspases CASP2/9, including the CARD domain and the p20/p10 subunits with conserved functional motifs. However, compared with human CASP2/9, AjCASPX1/2 possess unique structural features in the linker region between p20 and p10. AjCASPX1, but not AjCASPX2, induced marked apoptosis of human cells by activating CASP3/7. The recombinant proteins of AjCASPX2 and the CARD domain of AjCASPX2 were able to bind to a wide range of bacteria, as well as bacterial cell wall components, and inhibit bacterial growth. AjCASPX1, when expressed in *Escherichia coli*, was able to kill the host bacteria. Under normal conditions, AjCASPX1 and AjCASPX2 expressions were most abundant in sea cucumber muscle and coelomocytes, respectively. After bacterial infection, both AjCASPX1 and AjCASPX2 expressions were significantly upregulated in sea cucumber tissues and cells. Together, these results indicated that AjCASPX1 and AjCASPX2 were initiator caspases with antimicrobial activity and likely functioned in apoptosis and immune defense against pathogen infection.

## 1. Introduction

Apoptosis is involved in diverse biological processes, including growth, development, and immunity [1]. In mammals, two main pathways of apoptosis, i.e., the extrinsic pathway and the intrinsic pathway, have been identified [2]. The process of apoptosis is mediated by caspases (CASPs), a family of cysteine-aspartic specific proteases [3,4]. The CASPs are synthesized as inactive proenzymes consisting of an N-terminal pro-domain and a C-terminal catalytic domain [5]. The catalytic domain contains two subunits, the large (p20) and the small (p10) subunits, which can assemble into the active (p20)_2_(p10)_2_ heterotetramer [5,6]. Based on their functions and structures, CASPs can be divided into three subfamilies: apoptosis initiators (CASP2, 8, 9, and 10), apoptosis effectors (CASP3, 6, and 7), and inflammatory mediators (CASP1, 4, 5, 11, and 12) [7]. The pro-domain of initiator CASPs typically contains CASP recruitment domain (CARD) (in CASP2 and CASP9) or death effector domain (DED) (in CASP8 and CASP10), while the pro-domain of effector CASPs exhibits no structure [5,6]. The apoptosis initiators are activated by death signals; the activated initiators then activate the downstream effectors, which trigger apoptosis [1,8].

To date, at least 18 CASPs have been reported in mammals [9]. Among them, CASP2 and CASP9, which share similar structures, function mainly as apoptosis cascade mediators [10,11]. In the protease domain (CASc), the catalytic motifs of “SHG” and “QACRG” within the p20 region and the substrate binding motif of “GSWFI” within the p10 region are highly conserved [12,13]. The motif “SWR”, which is known to be involved in substrate binding of CASP3/7, is also present in the p10 subunit of CASP9, but not in the p10 subunit of CASP2 [10]. During the process of apoptosis, CASP2/9 is activated in the platform formed with CARD-containing proteins through CARD–CARD interactions [10,11]. CASP2 is activated in the PIDDosome, a platform containing PIDD1 (p53-induced death domain protein 1), to cleave the BH3-only protein BID, leading to mitochondrial membrane permeabilization, cytochrome c release, effector CASPs activation, and apoptosis [10]. In contrast, CASP9 can be recruited by apoptotic protease activating factor-1 (Apaf-1) to form an apoptosome that provides a platform for CASP9 dimerization and activation, which then activates CASP3/7 to induce apoptosis [11]. Additionally, several recent evidences demonstrated that CASP2 and CASP9 have non-apoptosis functions. For instance, CASP2 is considered a tumor suppressor in multiple cancer models [10,14], while CASP9 plays an important role in muscle differentiation [14,15].

The sea cucumber *A. japonicus* is an important economic species in East Asia, especially China. To date, several *A. japonicus* caspases have been identified and studied. Most of these studies focused on the expression of the caspases under different conditions. For example, one *A. japonicus* caspase (AjCASP1) was highly expressed in coelomocytes and respiratory tree under normal physiological conditions, and upregulated after *Vibrio splendidus* infection [16]. The expressions of other *A. japonicus* caspases were also stimulated by bacterial or lipopolysaccharide (LPS) challenge [17,18]. In the present work, we identified two previously unreported caspases from *A. japonicus* and examined their structure, apoptotic effect, and antimicrobial activity. Our results showed that, compared with their human counterparts, these two *A. japonicus* caspases possessed unique structural features. Functionally, one of the caspases possessed marked apoptosis-inducing activity, and both caspases exhibited direct antibacterial effects. These findings added new insight into the biological property and immune function of sea cucumber caspases.

## 2. Materials and Methods

### 2.1. Gene Cloning

Sea cucumbers *A. japonicus* (100 ± 15 g) were obtained from a local farm in Weihai (Shandong, China). In the laboratory, the sea cucumbers were kept in aerated seawater at 18 ± 1 °C for 1 to 2 weeks. Total RNA was extracted from sea cucumber tissues, including coelomocytes, intestine, muscle, and respiratory tree, using TRIzol reagent (Invitrogen, Carlsbad, CA, USA). cDNA was synthesized from total RNA (1 μg) with a cDNA Synthesis Kit (ToYoBo, Osaka, Japan). The coding sequences (CDSs) of AjCASPX1 and AjCASPX2 were amplified by PCR with primers based on the two caspases with GenBank accession numbers PIK40152.1 and PIK55213.1, respectively. The primers used are listed in Table S1.

### 2.2. Bioinformatics and Sequence Analysis

All caspase sequences used in the present study were obtained from the NCBI Database. The phylogenetic tree was conducted using maximum likelihood method with WAG+F+I+G4 model implemented in 1000 bootstrap replicates and imaged with iTOL (https://itol.embl.de/) (accessed on 26 January 2024) [19]. The multiple alignment of amino acid sequences was performed using the Jalview program (version: 2.11.3.0). The molecular weight and theoretical pI of caspases were calculated with the ProtParam (https://web.expasy.org/protparam/) (accessed on 26 January 2024). The structural domains of caspase-2/9 were analyzed by using the ScanProsite (https://prosite.expasy.org/scanprosite/) (accessed on 26 January 2024). The three-dimensional (3D) models of caspases were predicted with the Robetta website (http://robetta.bakerlab.org/) (accessed on 26 January 2024) and imaged using Visual Molecular Dynamics (VMD) (version: 1.9.3).

### 2.3. Transient Transfection

HEK293T cells were grown in DMEM supplemented with 10% FBS (Gibco, Renfrewshire, UK) at 37 °C in a 5% CO_2_ incubator. For transfection, HEK293T cells were seeded into 24-well plates (Corning, Corning city, NY, USA) at about 80% confluency. The CDSs of AjCASPX1 and AjCASPX2 were inserted into pCAGGS-Flag-N (Honor Gene, Changsha, China). Transient transfection was performed with PolyJet DNA transfection reagent (SignaGen, Ijamsville, MD, USA). After transfection for 12 h, 24 h, and 36 h, the bright-field views of transfected cells were taken with a fluorescence microscope (Nikon Corporation, Tokyo, Japan). The primers used are listed in Table S1.

### 2.4. Flow Cytometry

HEK293T cells were transfected as above for 36 h and then treated with Annexin V-FITC/Propidium iodide (PI) using an apoptosis detection kit (Beyotime biotechnology, Shanghai, China). Briefly, the cells were collected and resuspended in 195 µL binding buffer. Five microliters of Annexin V-FITC and 10 µL PI were successively added to the buffer, and the mixture were incubated for 15 min at 25 °C. The cells were immediately subjected to flow cytometry with FACSAria™ II (BD Biosciences, San Jose, CA, USA).

### 2.5. Lactate Dehydrogenase (LDH) Assay

HEK293T cells were transfected with the indicated plasmids as above. LDH releases at various time points were assessed with a cytotoxicity assay kit (Promega, Leiden, The Netherlands) according to the manufacturer’s instructions.

### 2.6. Immunoblotting

Immunoblotting was performed as described previously [20]. Briefly, the above transfected cells were lysed with lysis buffer (Beyotime, Shanghai, China). Proteins in lysate samples were separated by SDS-PAGE and transferred onto nitrocellulose membranes. After blocking with 5% skim milk in TBST for 1 h, the membranes were treated with mouse anti-Flag antibody or mouse anti-β-actin antibody (1:3000 dilution) at 4 °C overnight. The membranes were washed five times with TBST and then incubated with HRP-conjugated goat anti-mouse IgG (1:5000 dilution) for 1 h. The membranes were washed as above, and immune-reactive protein bands were detected with an enhanced chemiluminescence (ECL) kit (Sparkjade Biotechnology Co., Ltd., Jinan, China). All antibodies used were purchased from ABclonal (Wuhan, China).

### 2.7. Caspase Activity Analysis

The caspase activity in cells was examined as previously reported [21]. Briefly, the lysates were incubated with commercial fluorogenic substrates Ac-YVAD-AFC, Ac-VDVAD-AFC, Ac-DEVD-AFC, Ac-VEID-AFC, Ac-IETD-AFC, and Ac-LEHD-AFC (MedChem Express, Princeton, NJ, USA) in a 100 µL reaction system containing 50 mM HEPES (pH 7.5), 3 mM EDTA, 150 mM NaCl, 0.005% (*v*/*v*) Tween 20, and 10 mM DTT. Fluorescence intensity was detected every 5 min for 1 h with a BioTek Synergy HT plate reader (BioTek Instruments, Winooski, VT, USA).

### 2.8. Recombinant Protein Expression and Purification

Recombinant proteins were expressed and purified as described previously [22]. Briefly, the CDSs of AjCASPX2 variants were inserted into pET-30a (+) (Novagen, Madison, WI, USA). The recombinant plasmids were administered into *E. coli* BL21 (DE3) by transformation. The transformed bacteria were grown at 37 °C in LB broth with shaking. Recombinant proteins were induced with 0.1 mM IPTG and subsequent overnight growth at 16 °C. Bacterial cells were collected and lysed by ultrasonication on ice. Recombinant proteins in the supernatants were purified using Ni-NTA agarose (QIAGEN, Valencia, CA, USA) columns. The purified proteins were dialyzed in PBS and concentrated with an Ultrafree centrifugal filter (Millipore, Bedford, MA, USA).

### 2.9. Enzyme-Linked Immunosorbent Assay (ELISA) to Examine Protein–Bacteria Interaction

ELISA was performed as previously reported [23]. In brief, Gram-negative (*Vibrio anguillarum*, *Vibrio harveyi*, *Pseudomonas fluorescens*, *Edwardsiella piscicida*) and Gram-positive (*Streptococcus iniae*, *Micrococcus luteus*, *Bacillus subtilis*) bacteria were grown as described previously [24] and resuspended in coating buffer (0.159% Na_2_CO_3_, 0.293% NaHCO_3_, pH 9.6). Lipopolysaccharide (LPS) and peptidoglycan (PGN) (InvivoGen, San Diego, CA, USA) were diluted in coating buffer to a final concentration of 0.1 mg/mL. The bacterial resuspension or the LPS/PGN dilution was added into 96-well microtiter plates, and the plates were placed at 4 °C overnight. After blocking with 5% skim milk for 1 h, the plates were washed three times with PBST. Different concentrations (1, 2, 4, 8, 16, or 32 μg/mL) of AjCASPX2, CARD_AjCASPX2_, or Trx were added to the plates. The plates were incubated at 25 °C for 2 h and washed as above. The plates were then incubated with mouse anti-His tag antibody and HRP-conjugated goat anti-mouse IgG successively. The plates were washed five times with PBST, and TMB substrate solution (TIANGEN, Beijing, China) was added to the plates. The plates were analyzed with a microplate reader (BioTek Instruments, Winooski, VT, USA).

### 2.10. Effect of Caspase on Bacterial Growth

To examine the antimicrobial effect of AjCASPX2 variants, *V. harveyi* was diluted in LB medium to 2 × 10^8^ CFU/mL in a 96-well microtiter plate. One hundred microliters of AjCASPX2, CARD_AjCASPX2_, or Trx protein solution (final concentration 32 μg/mL) were placed into the plate. The bacterial cells were grown at 28 °C with shaking (180 rpm), and the bacterial growth was recorded every hour by measuring OD_600_. The effect of AjCASPX1 expression on host bacteria was conducted as previously reported [23]. Briefly, *E. coli* BL21 (DE3) carrying AjCASPX1 gene on pET-30a was grown in LB broth to OD_600_ 0.6. The cells were diluted and grown on LB agar plates supplemented with or without 0.3 mM IPTG to induce the expression of AjCASPX1. The plates were cultured at 37 °C overnight, and colony forming units (CFUs) on the plates were counted and statistically calculated.

### 2.11. Quantitative Real Time PCR (qRT-PCR)

AjCASPX1/2 expressions in sea cucumber tissues were examined by qRT-PCR with a ChamQ SYBR qPCR Master Mix (Vazyme Biotech, Nanjing, China) as previously reported [21]. The expression levels of AjCASPX1 and AjCASPX2 were analyzed using the comparative threshold cycle method [2^-(∆∆Ct)^], with the mRNA levels normalized to that of β-actin. The primers used for qRT-PCR are listed in Table S1.

### 2.12. Bacterial Infection

For in vivo infection, the pathogen *V. harveyi* was collected and resuspended in PBS to a final concentration of 5 × 10^8^ CFU/mL. Thirty sea cucumbers were randomly divided into two groups and injected with 100 μL *V. harveyi* and PBS, respectively. At 6 h, 24 h, and 48 h post infection (hpi), tissues were collected aseptically. For in vitro infection, *V. harveyi* was resuspended in L15 medium (Sigma Aldrich, Madrid, Spain). The coelomocytes were collected from sea cucumber as reported previously [25]. Briefly, the coelomic fluids were extracted and filtered through 40 μm cell strainers, and then mixed with an equal volume of anticoagulant solution (0.02 M EDTA, 0.48 M NaCl, 0.019 M KCl, and 0.068 M Tri-HCl, pH 7.6). After centrifugation at 800× *g* at 4 °C for 5 min, coelomocytes were collected and resuspended in L15 medium with 0.39M NaCl. Coelomocytes were infected with or without (control) *V. harveyi* (MOI = 5) for 3 h or 6 h. Gene expression was then determined by qRT-PCR.

### 2.13. Statistical Analysis

All statistical analyses were performed with GraphPad Prism 8. The significance between groups was analyzed with Student’s *t*-test and one-way ANOVA. Statistical significance was defined as * *p* < 0.05.

## 3. Results

### 3.1. Characterization of Two Caspases from A. japonicus

**Figure 1:**
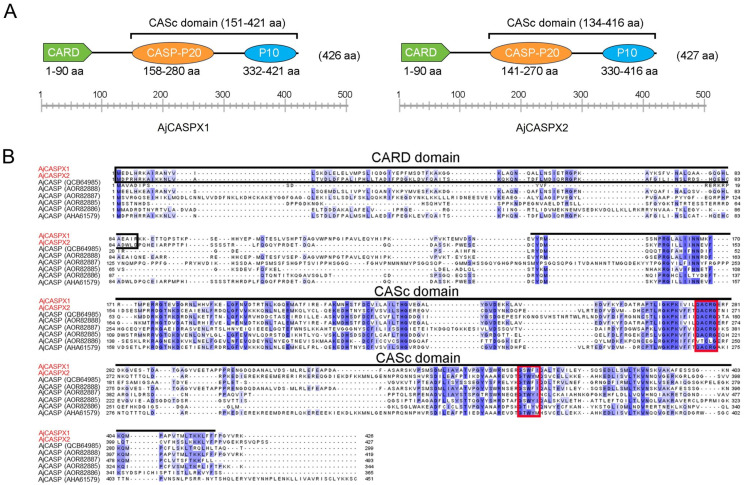
Sequence analysis of sea cucumber caspases. (**A**) The structural domains of AjCASPX1 and AjCASPX2, with the CARD, CASP-p20, and CASP-p10 colored in green, orange, and light blue, respectively. (**B**) Sequence alignment of AjCASPX1/2 and the reported sea cucumber CASPs. AjCASPX1/2 is highlighted in red. The residues that are >50% identical are shaded, with identical residues shaded in dark blue. The active-site pentapeptide motif “QACRG” and the protein binding motif “GSWFI” are marked with red boxes.

The coding sequences of two caspases, named AjCASPX1 and AjCASPX2, respectively, were cloned based on the genome sequence of *A. japonicus*. AjCASPX1 contains 426 amino acid residues, with a calculated molecular weight of 47.52 kDa and a theoretical pI of 6.58. AjCASPX2 consists of 427 amino acid residues, with a calculated molecular weight of 49.17 kDa and a theoretical pI of 5.91. Both caspases exhibit a caspase recruitment (CARD) domain and a CASc domain containing the p20 and p10 subunits (Figure 1A). AjCASPX1 and AjCASPX2 are 28.25% identical with each other and share 18.15–82.86% and 14.60–76.94% sequence identities, respectively, with the reported sea cucumber caspases. The active-site pentapeptide “QACRG” of mammalian caspases is highly conserved in AjCASPX1/2 (Figure 1B). The substrate binding motif “GSWFI” is completely conserved in AjCASPX1 but less conserved in AjCASPX2 and other sea cucumber caspases (Figure 1B). Phylogenetic analysis with mammalian caspases showed that AjCASPX1 and AjCASPX2 were relatively close to the CARD-containing initiator caspases (CASP2/9) and clearly excluded from the clades of DED-containing initiator caspases (CASP8/10) and the effector caspases (CASP3/7/6) (Figure S1).

### 3.2. AjCASPX1 and AjCASPX2 Possess Unique Structural Features in the CASc Domain

**Figure 2:**
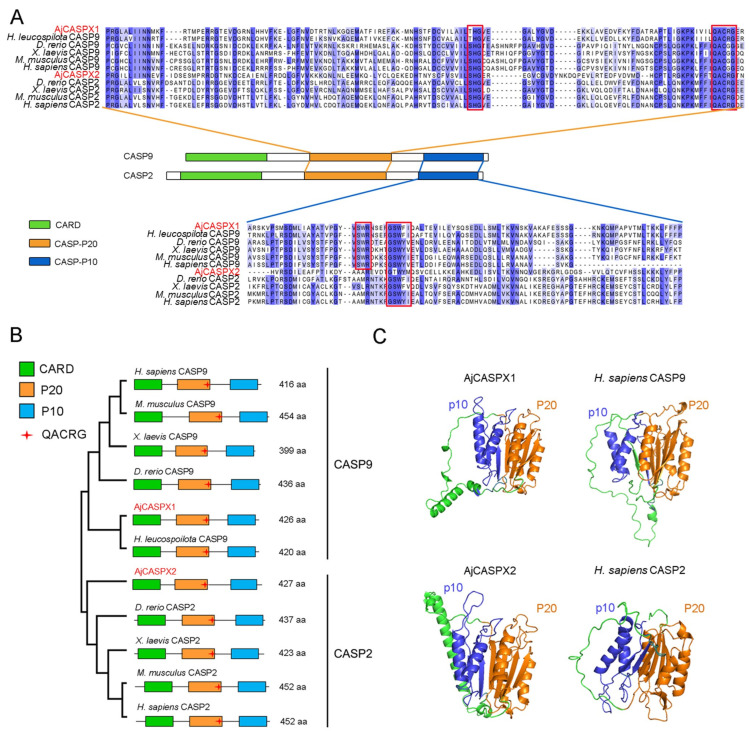
Comparative analysis of AjCASPX1/2 with caspase-2/9. (**A**) Sequence alignment of the p20 and p10 regions of confirmed CASP9 and CASP2 in various species. AjCASPX1/2 are highlighted in red. The residues that are >50% identical are shaded, with identical residues shading in dark blue. The key motifs of CASPs in p20 and p10 regions are marked with red boxes. (**B**) The structural organization of the above CASP9 and CASP2. (**C**) Comparison of the predicted 3D structures of the CASc domains of AjCASPX1/2 and *Homo sapiens* CASP2/9.

Comparative sequence analyses of AjCASPX1/2 and well-studied CASP2/9 from different species indicated that AjCASPX1, like all CASP9, possessed a conserved “SWR” motif in the p10 subunit, whereas AjCASPX2, like all CASP2, lacked the “SWR” motif in p10 (Figure 2A). The general structural organizations, including the CARD domain, the p20/p10 subunits, and the nestled pentapeptide “QACRG”, of AjCASPX1 and AjCASPX2 were similar to that of CASP2/9 (Figure 2B,C). However, AjCASPX1 differed from human CASP9 in the three-dimensional (3D) structure of the CASc domain by exhibiting an α-helix between p20 and p10 (Figure 2C). Similarly, compared with human CASP2, AjCASPX2 displayed an extra α-helix between p20 and p10.

### 3.3. AjCASPX1 Is Activated and Induces Apoptosis in HEK293T

**Figure 3:**
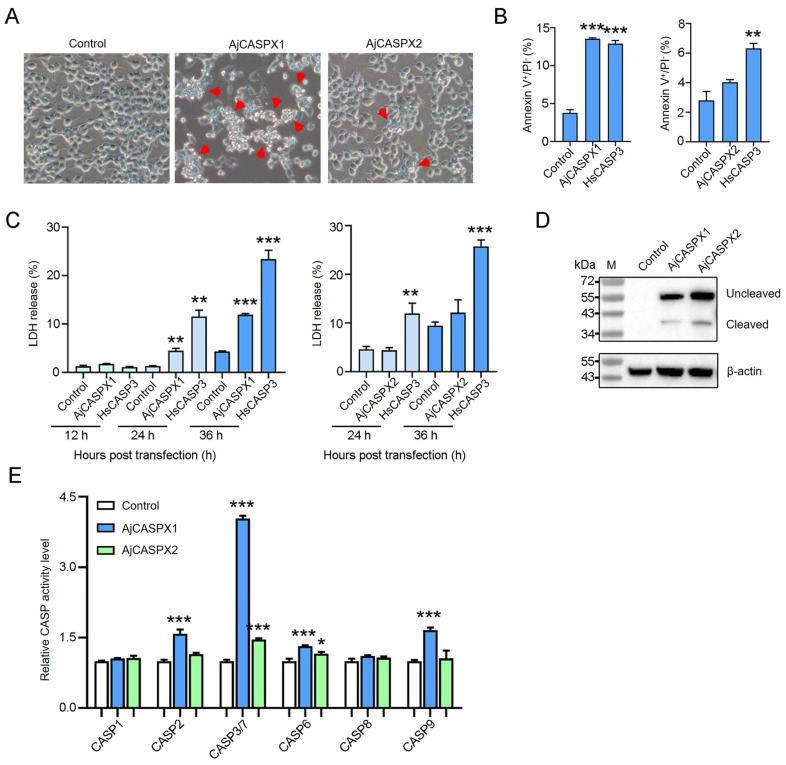
Apoptosis-inducing activity of AjCASPX1 and AjCASPX2. (**A**) HEK293T cells were transfected with the backbone vector (control) or the vector expressing Flag-tagged AjCASPX1 or AjCASPX2 for 36 h and then observed for morphological change. Red arrows indicate apoptotic cells. (**B**) HEK293T cells were transfected as above. The positive control cells were transfected with the vector expressing human caspase-3 (HsCASP3). The cells were stained with Annexin V–FITC and PI and subjected to flow cytometry. The percentages of Annexin V^+^/PI^−^ cells were statistically calculated. (**C**) HEK293T cells were transfected as above with indicated vectors for various time and monitored for LDH release. (**D**,**E**) HEK293T cells transfected with indicated vectors were blotted with antibody against Flag or β-actin (**D**). The CASP 1/2/3/7/6/8/9 activities in the cell lysate were determined (**E**). For panels (**B**,**C**,**E**), values are the means of three experimental replicates and shown as means ± SD. *** *p* < 0.001; ** *p* < 0.01; * *p* < 0.05.

To determine the apoptosis-inducing activity of AjCASPX1 and AjCASPX2, Flag-tagged AjCASPX1 and AjCASPX2 were each expressed in HEK293T cells. Microscopy showed that AjCASPX1 induced strong apoptosis, while AjCASPX2 induced only slight apoptosis (Figure 3A). In AjCASPX1-expressing cells, the percentage of the Annexin V^+^/PI^−^ cells significantly increased compared with that of the control cells, whereas in cells expressing AjCASPX2, no significant change in Annexin V^+^/PI^−^ cells was observed (Figure 3B). Following transfection of HEK293T, AjCASPX1 induced no or little LDH release in the early stage, but triggered abundant LDH release in the late stage (Figure 3C). In contrast, AjCASPX2 induced no apparent LDH release in either the early or the late stage. Immunoblotting analysis revealed that both AjCASPX1 and AjCASPX2 were cleaved and activated in HEK293T cells (Figure 3D). Furthermore, in AjCASPX1-expressing cells, CASP3/7 activities markedly and significantly elevated, while CASP2/6/9 activities increased slightly but significantly (Figure 3E). In AjCASPX2-expressing cells, only CASP3/7/6 activities increased significantly, although to a slight extent (Figure 3E).

### 3.4. AjCASPX2 and AjCASPX1 Exhibit Antibacterial Activity

**Figure 4:**
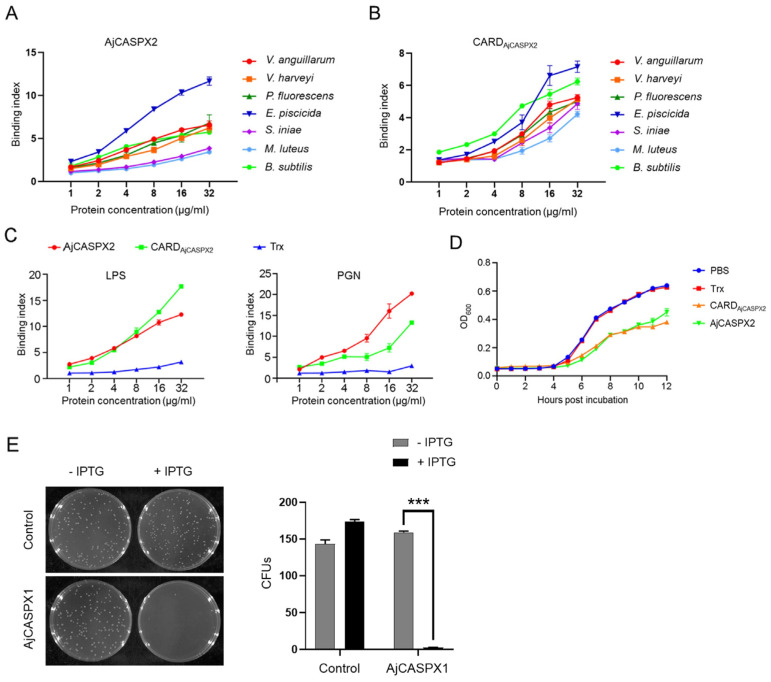
Antibacterial effects of AjCASPX1/2. (**A**,**B**) Bacteria were incubated with different concentrations of AjCASPX2 (**A**) and CARD_AjCASPX2_ (**B**) for 2 h, and binding activity was assessed by ELISA. (**C**) AjCASPX2, CARD_AjCASPX2_, or Trx in different concentrations were incubated with LPS or PGN, and the binding was assessed by ELISA. (**D**) *V. harveyi* was incubated with AjCASPX2, CARD_AjCASPX2_, Trx, or PBS, and the bacterial growth was determined. (**E**) *E. coli* was transformed with the backbone vector (control) or the vector carrying the AjCASPX1 gene. The cells were plated in LB agar plates supplemented with or without IPTG and cultured overnight. The number of survived bacteria were determined (right panel). For all panels, values are shown as means ± SD. n = 3. *** *p* < 0.001.

To examine its biological activity, AjCASPX2 was purified from *E. coli* as a recombinant protein (Figure S2). As observed with many recombinant caspases which have undergone activation by auto-cleavage during purification [21,26], auto-cleavage of recombinant AjCASPX2 also occurred (Figure S2). AjCASPX2 bound to Gram-negative (*V. anguillarum*, *V. harveyi*, *P. fluorescens*, and *E. piscicida*) and Gram-positive (*S. iniae*, *M. luteus*, and *B. subtilis*) bacteria in a dose-dependent pattern (Figure 4A). The recombinant protein of the N-terminal CARD domain of AjCASPX2 (CARD_AjCASPX2_) showed similar binding patterns to these bacteria (Figure 4B). Both AjCASPX2 and CARD_AjCASPX2_ exhibited strong binding abilities to lipopolysaccharide (LPS) and peptidoglycan (PGN) in a dose-dependent manner (Figure 4C). For *V. harveyi*, a common pathogen to sea cucumber and many other marine animals, its growth was apparently hampered by AjCASPX2 and CARD_AjCASPX2_ (Figure 4D). Unlike AjCASPX2, AjCASPX1 could not be purified from *E. coli* as a recombinant protein. When AjCASPX1 was induced to express in the host *E. coli* cells, almost no cell survival was observed (Figure 4E), implying a bactericidal effect of AjCASPX1.

### 3.5. AjCASPX1 and AjCASPX2 Are Involved in V. harveyi Infection

**Figure 5:**
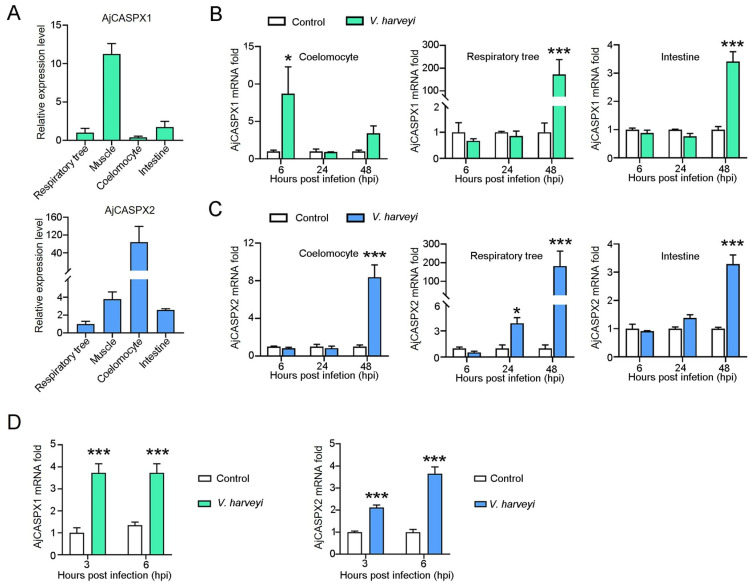
AjCASPX1/2 expression in the presence and absence of bacterial infection. (**A**) AjCASPX1 and AjCASPX2 expressions in sea cucumber tissues were analyzed by qRT-PCR. The expression levels are presented relative to that in the respiratory tree. (**B**,**C**) Sea cucumbers were incubated with or without (control) *V. harveyi*, and the expression of AjCASPX1 (**B**) and AjCASPX2 (**C**) in tissues was determined by qRT-PCR. (**D**) Sea cucumber coelomocytes were treated with or without (control) *V. harveyi* for 3 h and 6 h, and the expression of AjCASPX1 and AjCASPX2 was analyzed. For all panels, values are the means of three experimental replicates and shown as means ± SD. *** *p* < 0.001; * *p* < 0.05.

qRT-PCR showed that AjCASPX1 and AjCASPX2 expressions were detected in respiratory tree, muscle, coelomocytes, and intestine under normal physiological conditions (Figure 5A). The highest expression levels of AjCASPX1 and AjCASPX2 occurred in muscle and coelomocytes, respectively. When the sea cucumbers were infected with *V. harveyi*, AjCASPX1 expression significantly increased at 6 hpi in coelomocytes and at 48 hpi in respiratory tree and intestine, while AjCASPX2 expression significantly increased at 48 hpi in all three tissues (muscle, coelomocytes, and respiratory tree) (Figure 5B,C). Cellular analysis showed that when the isolated coelomocytes were incubated with *V. harveyi*, AjCASPX1 and AjCASPX2 expressions significantly increased at 3 and 6 hpi (Figure 5D).

## 4. Discussion

The caspase superfamily contains plentiful members belonging to different subfamilies, many of which have been documented to participate in apoptosis [2,5,27]. CASP2 and CASP9 are two members of the apoptosis initiator CASP subfamily. Up to now, only a small number of invertebrate CASP2/9 have been examined for their roles in apoptosis signaling [17,28,29]. In the present study, we identified and characterized two CASPs, AjCASPX1 and AjCASPX2, from sea cucumber *A. japonicus*. AjCASPX1 and AjCASPX2 possess the characteristic motifs conserved in all caspases, such as QACRG and GSWFI, which are crucial for catalytic reaction and substrate binding [12,13]. Since the classification of caspases are based on mammalian caspases, we compared the sequence and structure of AjCASPX1/2 with human and mouse caspases. We found that phylogenetically AjCASPX1/2 were relatively closely related to mammalian CASP2/9. Consistently, the tripeptide motif SWR in the small catalytic subunit p10, which is known to be involved in substrate binding of some CASPs [12,13,30], was present in the well-studied CASP9 and in AjCASPX1, but not present in the well-studied CASP2 or AjCASPX2. These results indicated that AjCASPX1 and AjCASPX2 were initiator caspases, likely belonging to the mammalian CASP9 and CASP2 lineages, respectively. However, although AjCASPX1 and AjCASPX2 have a general domain organization similar to that of human CASP2/9, there is a marked difference between AjCASPX1/2 and human CASP2/9 in the linker region connecting the p20 and p10 subunits. The extra α-helix in this region of AjCASPX1/2 suggests a functional or regulation difference between AjCASPX1/2 and mammalian CASP2/9.

To date, the biological functions of invertebrate CASP2/9 remain largely unknown. Of the few reported invertebrate CASP2/9 with functional analysis, the CASP2 of mud crab *Scylla paramamosain* and the CASP9 of sea cucumber *Holothuria leucospilota* were able to augment apoptosis induction [28,29]. In our study, we found that AjCASPX1 exhibited marked ability to trigger apoptosis of HEK293T cells, and the process was accompanied by significant activation of CASP3/7, implying that AjCASPX1 functioned as an initiator caspase that cleaved the effector caspases CASP3/7, which further induced apoptotic cell death. In contrast to AjCASPX1, AjCASPX2 was a very weak apoptosis inducer in human cells, possibly due to its ineffectiveness to activate the human effector molecules, as evidenced by the very slight, though significant, CASP3/7 activation in AjCASPX2-expressing cells. In addition, since there are evidences showing that CASP2-induced apoptosis in mammals was independent of CASP3/7 but required the activity of PIDD1 [31,32,33], it is possible that AjCASPX2-mediated apoptosis might be limited to certain conditions that requires the presence of some specific proteins.

Previous studies showed that some mammalian inflammatory caspases, such as human CASP4/5 and mouse CASP11, bind LPS directly through their CARD domains [34,35]. In invertebrates, the CASP1 of sea cucumber *A. japonicus* and oyster *Crassostrea gigas* have been reported to possess LPS binding capacity [16,36]. In the present study, we found that AjCASPX2 interacted directly with a wide range of Gram-positive and Gram-negative bacteria. These interactions were further supported by the apparent binding activities of AjCASPX2 towards the cell wall components of Gram-positive and Gram-negative bacteria, i.e., LPS and PGN. Since, as observed in previous reports with many caspases [21,26], the CARD domain was removed via auto-processing in purified AjCASPX2, the bacteria-binding capacity of AjCASPX2 was CARD-independent, which was unlike that observed in mammalian inflammatory caspases [34,35]. It is interesting that the CARD domain of AjCASPX2 was also capable of bacteria and LPS/PGN binding. These results suggested a potent bacteria interaction ability of AjCASPX2 mediated by both the N-terminal CARD and the C-terminal CASc. Since the binding of AjCASPX2 as well as CARD_AjCASPX2_ significantly inhibited the growth of *V. harveyi*, AjCASPX2 likely functioned as an antimicrobial factor against bacterial pathogens. It is also possible that AjCASPX2 might act as a receptor that senses bacteria to activate the downstream cascade signaling. Like AjCASPX2, AjCASPX1 also exhibited antimicrobial activity, as it exerted a lethal effect on the host bacterial cells in which it was expressed, suggesting that AjCASPX1 possessed bactericidal property.

In mammals, CASPs have been shown to be extensively involved in the immune defense induced by pathogenic microorganisms [37,38,39]. In this study, we found that under normal physiological conditions, AjCASPX1 was predominately expressed in muscle, which is the main part of body wall undergoing massive tissue autolysis [40]. A previous report showed that apoptosis was an essential and indispensable process in sea cucumber autolysis [41]. The high expression level of AjCASPX1 in muscle suggested a possible role of AjCASPX1 in autolysis. AjCASPX2 was most abundantly expressed in the coelomocytes, which are considered the main sea cucumber immune cells with phagocytosis ability [42]. Given the strong bacteria binding capacity of AjCASPX2, it is likely that various bacteria might be pooled by coelomocytes, where they are recognized and bound by AjCASPX2, resulting in inhibition of bacterial growth and invasion. In line with their antibacterial capacity, AjCASPX1 and AjCASPX2 were significantly upregulated in expression in *V. harveyi*-challenged sea cucumbers and coelomocytes, further supporting the role of AjCASPX1 and AjCASPX2 in antimicrobial immune defense.

## 5. Conclusions

In conclusion, our study demonstrated that AjCASPX1 and AjCASPX2 were initiator caspases that may be of the mammalian CASP9 and CASP2 lineages, respectively; however, AjCASPX1/2 possessed distinct structural features that may enable them to function in a manner different from that of mammalian CASP2/9. The apoptotic activity of AjCASPX1 and the antibacterial effects of AjCASPX2 and AjCASPX1 suggested a role of these caspases in apoptosis and immune defense against bacterial infection. These findings may be applied to the development of better culturing methods and disease control approaches for sea cucumber in aquaculture.

## 6. Limitations of the Study

In this study, we examined the biological activity of AjCASPX1 and AjCASPX2 under *in vitro* conditions utilizing the HEK293T cells and *E. coli*. While the HEK293T cell line is a common and mature model for the study of invertebrate genes, the results thus obtained may not reflect the activity of the examined gene under *in vivo* condition. For eukaryotic proteins, their recombinant proteins expressed in *E. coli* may exhibit properties different from that of the native proteins due to structural difference. Therefore, future studies are needed to examine whether the apoptotic and antibacterial activities of AjCASPX1/2 observed in this study reflect the native function of these caspases in sea cucumber.

## Data Availability

All data in the paper are present in the paper or the Supplementary Materials.

## Supplementary Materials

The following supporting information can be downloaded at: https://www.mdpi.com/article/10.3390/genes15050540/s1, Figure S1: Phylogenetic analysis of AjCASPX1/2 with human and mouse caspases; Figure S2: Preparation of recombinant proteins; Table S1: Primers used in this study.
