# Supplementary material for: Characterization of the Apoptotic and Antimicrobial Activities of Two Initiator Caspases of Sea Cucumber Apostichopus japonicus
Source: Genes (Basel). 2024 Apr 25;15(5):540. doi: 10.3390/genes15050540 (PMC11121444; doi:10.3390/genes15050540)

Supplementary Materials

This file include:

Table S1. Primers used in this study.

Figure S1. Phylogenetic analysis of AjCASPX1/2 with human and mouse caspases.

Figure S2. Preparation of recombinant proteins.

Table S1. Primers used in this study.

Primers	Sequence (5'–3')
**For gene cloning**	
AjCASPX1 forward	ATGGAGGACCTTCACAGGA
AjCASPX1 reverse	TCATTTTCTTACATAACCAGGAAAG
AjCASPX2 forward	ATGGACCCACGGCACAG
AjCASPX2 reverse	TTAGCATGATTTCTTGTAGAGGCATG
**For expression in HEK293T**	
AjCASPX1-pCAGGS-N-FLAG forward	GATGACGACGATAAGGAATTCATGGAGG ACCTTCACAGGA
AjCASPX1-pCAGGS-N-FLAG reverse	AATTAATTAAGATCTGCTAGCTCATTTTCT TACATAACCAGGAAAG
AjCASPX2-pCAGGS-N-FLAG forward	GATGACGACGATAAGGAATTCATGGACCC ACGGCACAG
AjCASPX2-pCAGGS-N-FLAG reverse	AATTAATTAAGATCTGCTAGCTCAAGAGG ATGGTTGTACTGAACGTTTC
**For expression in *E. coli***	
AjCASPX1-pET30a(+) forward	TAAGAAGGAGATATACATATGGGTGTCTG GCCAAATCC
AjCASPX1-pET30a(+) reverse	GTGGTGGTGGTGGTGCTCGAGTTTTCTTA CATAACCAGGAAAG
AjCASPX2 -pET30a(+)forward	TAAGAAGGAGATATACATATGCAATATCCA AGAGATGAGACAGATC
AjCASPX2 -pET30a(+) reverse	GTGGTGGTGGTGGTGCTCGAGAGAGGAT GGTTGTACTGAACGTTTC
$CARD_{AjCASPX2}$ -pET30a(+)forward	TAAGAAGGAGATATACATATGATGGACCC ACGGCACAG
$CARD_{AjCASPX2}$-pET30a(+) reverse	GTGGTGGTGGTGGTGCTCGAGACTTGCA

	TCTTGACCAGCCT
**For Real-time PCR expression analysis**	
AjCASPX1-RT forward	TTCAAGGCCAAAGGTGGGAAG
AjCASPX1-RT reverse	CAGCATCAGTCGGGTGTGAA
AjCASPX2-RT forward	CACAGCATGAAATTGCGCGT
AjCASPX2-RT reverse	TCTCGCCTGGGCATTGATTC
Ajβ-actin-RT forward	CCATTCAACCCTAAAGCCAACA
Ajβ-actin-RT reverse	ACACACCGTCTCCTGAGTCCAT

Figure S1. Phylogenetic analysis of AjCASPX1/2 with human and mouse caspases. The phylogenetic tree was constructed with WAG+F+I+G4 model implement in IQ-TREE 2 v.2.1.2. The values at the forks indicate the percentage of trees in which this grouping occurred after bootstrapping (1000 replicates). Scale bar, the number of substitutions per base. The accession numbers of the listed protein sequences are as follows: *Homo sapiens* CASP1 (NP_001244047.1), *Mus musculus* CASP1 (NP_033937.2), *Homo sapiens* CASP2 (NP_116764.2), *Mus musculus* CASP2 (NP_031636.1), *Homo sapiens* CASP3 (XP_003823509.1), *Mus musculus* CASP3 (NP_001271338.1), *Homo sapiens* CASP4 (NP_001216.1), *Mus musculus* CASP4 (NP_001366247.1), *Homo sapiens* CASP5 (NP_004338.3), *Homo sapiens* CASP6 (NP_001217.2), *Mus musculus* CASP6 (NP_033941.3), *Homo sapiens* CASP7 (NP_001218.1), *Mus musculus* CASP7 (NP_031637.1), *Homo sapiens* CASP8 (NP_001073593.1), *Mus musculus* CASP8 (NP_001073595.1), *Homo sapiens* CASP9 (NP_001220.2), *Mus musculus* CASP9 (NP_056548.2), *Homo sapiens* CASP10 (NP_116756.2), *Mus musculus* CASP11 (CAA73531.1)

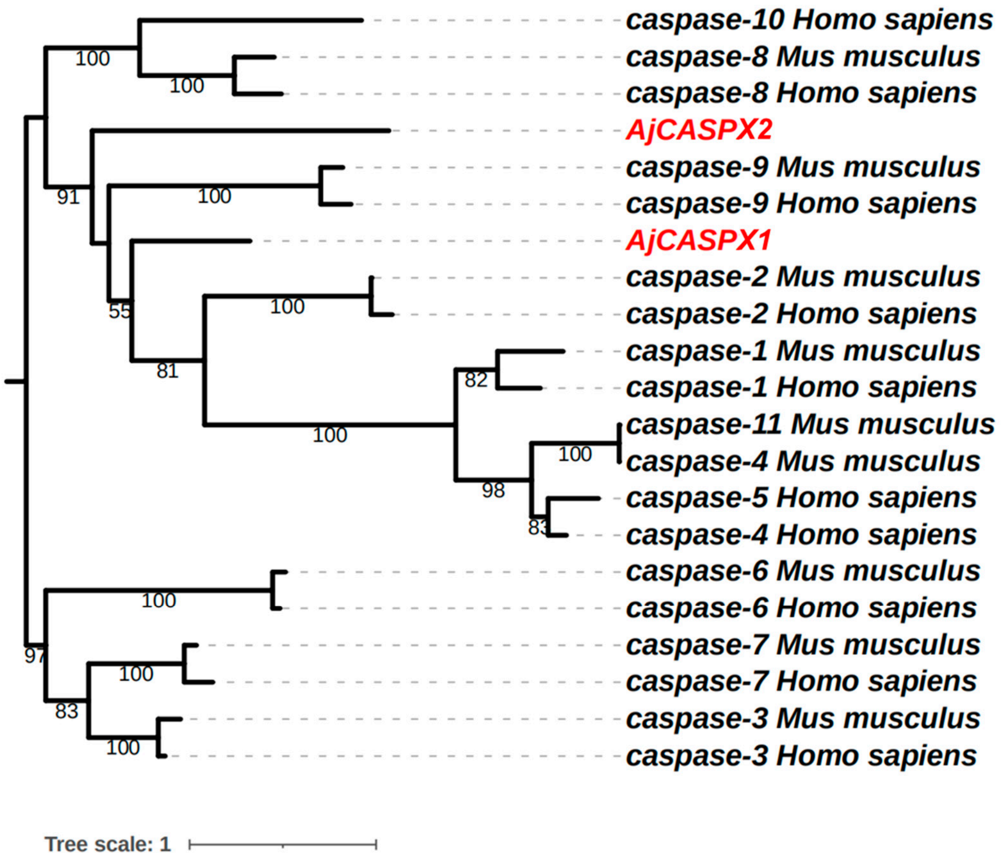
caspase-10 Homo sapiens
caspase-8 Mus musculus
caspase-8 Homo sapiens
AjCASPX2
caspase-9 Mus musculus
caspase-9 Homo sapiens
AjCASPX1
caspase-2 Mus musculus
caspase-2 Homo sapiens
caspase-1 Mus musculus
caspase-1 Homo sapiens
caspase-11 Mus musculus
caspase-4 Mus musculus
caspase-5 Homo sapiens
caspase-4 Homo sapiens
caspase-6 Mus musculus
caspase-6 Homo sapiens
caspase-7 Mus musculus
caspase-7 Homo sapiens
caspase-3 Mus musculus
caspase-3 Homo sapiens
100
100
91
100
55
100
81
82
100
100
98
83
100
97
100
83
100
Tree scale: 1

Figure S2. Preparation of recombinant proteins. SDS-PAGE analysis of purified AjCASPX2 (A) and $CARD_{AjCASPX2}$ (B).

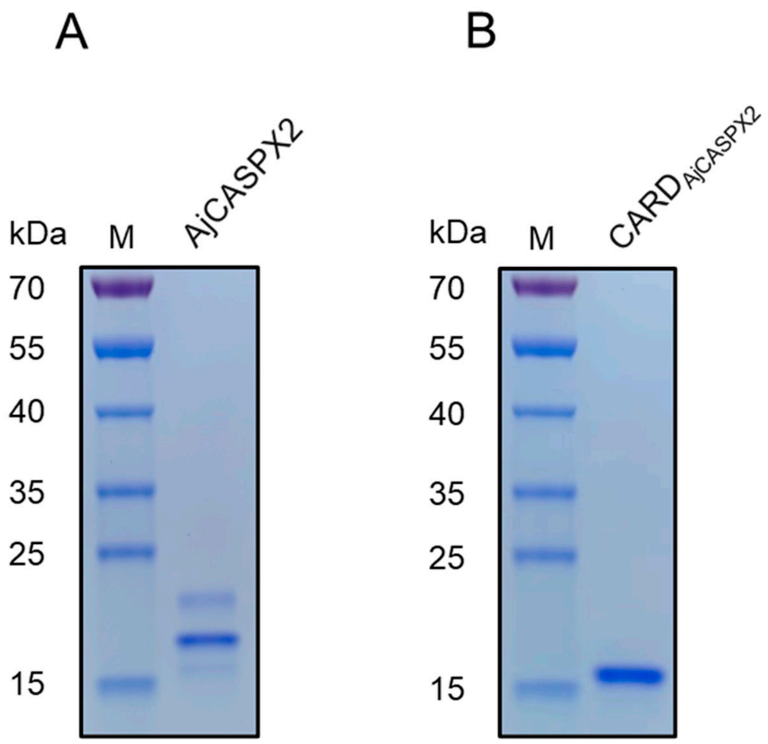

Supplement: Supplementary file 1 [file genes-15-00540-s001.zip › genes-2956298-supplementary.pdf]
